# Why Every Asthma Patient Tells a Different Story

**DOI:** 10.3390/jcm14165641

**Published:** 2025-08-09

**Authors:** Alessio Marinelli, Silvano Dragonieri, Andrea Portacci, Vitaliano Nicola Quaranta, Giovanna Elisiana Carpagnano

**Affiliations:** Department of Respiratory Diseases, University of Bari, 70121 Bari, Italy; alessio.marinelli@uniba.it (A.M.); a.portacci01@gmail.com (A.P.); vitalianonicola.40@gmail.com (V.N.Q.); elisiana.carpagnano@uniba.it (G.E.C.)

**Keywords:** asthma, perspective, pulmonology, asthma heterogeneity, endotypes, precision medicine

## Abstract

Asthma has traditionally been viewed as a single disease, but recent research reveals its clinical and molecular complexity. This perspective highlights the need to shift from a traditional, uniform treatment paradigm to one that embraces the heterogeneity of asthma across individuals. Each patient presents a unique clinical story shaped by a complex interplay of genetic predispositions, developmental programming during critical early-life windows, the influence of sex and hormones, and lifelong environmental exposures. Asthma comprises multiple subtypes with distinct clinical and biological features. Furthermore, lifestyle factors such as obesity and smoking, along with highly prevalent comorbidities like allergic rhinitis and gastroesophageal reflux disease, significantly modify the disease’s course and response to treatment. This article explores how classifying the disease into clinical phenotypes (observable characteristics) and molecular endotypes (underlying mechanisms)—particularly the distinction between T2-high and T2-low inflammation—provides a crucial framework for managing this complexity. The application of this framework, guided by biomarkers, has enabled the development of targeted biologic therapies that can transform care for specific patient subgroups. Despite these advances, significant challenges remain. The pathophysiology of certain subgroups, particularly non-T2 asthma, remains poorly defined, and there is an urgent need for reliable predictive biomarkers to guide therapy and monitor outcomes. It is our opinion that future studies must adopt a systems-biology strategy, with a multi-omics approach that constructs a comprehensive molecular profile of each patient. This integrative methodology will require the use of advanced computational methods, including machine learning and artificial intelligence, to decipher the complex pathways linking genetic and environmental inputs to clinical disease. In conclusion, this article argues for a more personalized understanding of asthma, urging clinicians and researchers to consider each patient’s unique clinical presentation.

## 1. Introduction

Asthma has long been recognized as a major global health issue, characterized by recurrent episodes of wheezing, shortness of breath, chest tightness, and coughing [1]. Historically, it was often treated as a single disease defined by a common set of symptoms and signs of reversible airway obstruction [2]. In recent years, however, it has been accepted that asthma is a heterogeneous syndrome comprising multiple disorders with shared clinical features but distinct underlying mechanisms [2,3].

In this perspective, we aim to explore the complexity of asthma, which arises from the unique constellation of genetic predispositions, developmental influences, environmental exposures, and clinical characteristics present in each patient. Physicians must move beyond the traditional uniform treatment paradigm, which has dominated asthma management for years, as it is proving increasingly inadequate for a significant portion of patients who remain uncontrolled despite adherence to standard guidelines [3,4]. This reflects a failure to align therapies with the patient’s specific pathophysiological profile.

### 1.1. Epidemiology

Asthma represents a substantial public health challenge worldwide and is recognized as a major noncommunicable disease (NCD) with a significant and varied global impact [5]. As the most common chronic disease in children and a major cause of morbidity in adults, its global prevalence is estimated to be between 262 and 339 million people [6,7,8]. The annual mortality is approximately 460,000, making it the second leading cause of death among chronic respiratory diseases [7]. In the United States, 2023 data show a prevalence of 8.9% in adults and 6.7% in children, resulting in millions of clinical consultations annually [9]. Similarly, in the European Union, the prevalence is estimated at 8.2% in adults and 9.4% in children [10].

However, significant regional and national disparities exist in the disease’s burden. An analysis from the Global Burden of Disease (GBD) study reveals a complex pattern (Table 1) [7]. This discrepancy highlights a crucial “global asthma paradox” which is clearly illustrated by contrasting age-standardized rates (ASR) across different regions. The ASR adjusts the data to what it would be if all regions had the same standard age structure, which allows for meaningful comparisons of health data across different regions of the world.

- **High Prevalence, Low Mortality:** High-income regions like North America have one of the highest asthma prevalence rates in the world (ASR of 9717.74 per 100,000). For instance, Western Europe reports one of the lowest mortality rates (ASR of 0.70 per 100,000).
- **Lower Prevalence, High Mortality:** Conversely, regions such as South Asia experience a disproportionately high mortality burden (ASR of 17.68 per 100,000), while Oceania has the highest death rate of all (ASR of 33.98 per 100,000). Over 80% of all asthma-related deaths occur in low- and middle-income countries (LMICs) [6].

**Table 1:** Global and Regional Asthma Epidemiology. This table illustrates the “Global Asthma Paradox” where regions with the highest prevalence (e.g., high-income North America) have low mortality, while regions with lower prevalence (e.g., South Asia) or small populations (e.g., Oceania) bear a disproportionately high mortality and disability burden [7]. ASR, age-standardized rate per 100,000 people.

Indicator	Location	ASR
Incidence	High-income North America	1403.64
	Caribbean	1193.84
	Central Europe	898.73
Prevalence	High-income North America	9717.74
	Australasia	7747.21
	Caribbean	7638.48
Deaths	Oceania	33.98
	South Asia	17.68
	Central Sub-Saharan Africa	15.79
DALYs	Oceania	847.59
	Central Sub-Saharan Africa	491.68
	Caribbean	468.60

The high prevalence in developed nations suggests that factors associated with Westernized lifestyles, such as urbanization and the “hygiene hypothesis”, are potent drivers of asthma development. Conversely, the disease’s lethality is highest in regions with lower socioeconomic status. This is attributable not to a greater intrinsic severity of the disease, but to systemic failures in healthcare infrastructure. In many LMICs, under-diagnosis, under-treatment, and a lack of access to essential controller medications like inhaled corticosteroids (ICS) are widespread, transforming a manageable chronic condition into a life-threatening one [11]. Consequently, while the clinical journey for a patient in a high-income country is typically focused on symptom management, for a patient in an LMIC, it is often a matter of survival against a backdrop of inadequate care.

### 1.2. Asthma Diagnosis and Management

The Global Initiative for Asthma (GINA) strategy, which is updated annually, provides the evidence-based foundation for asthma diagnosis and management globally [1,4]. GINA defines asthma as “a heterogeneous disease, usually characterized by chronic airway inflammation”, thereby anchoring its diagnosis in underlying pathology rather than symptoms alone [1]. A formal diagnosis requires both a history of variable respiratory symptoms (e.g., wheeze, cough) and confirmed variable expiratory airflow limitation. Objective confirmation is crucial and can be established via spirometry (post-bronchodilator increase in FEV1 of ≥12% and ≥200 mL), significant variability in twice-daily peak expiratory flow (PEF), or a positive bronchial challenge test.

A central evolution in the GINA strategy has been the shift away from reliance on short-acting beta-agonist (SABA) relievers for all patients. This change is driven by strong evidence linking SABA overuse to an increased risk of severe exacerbations and mortality, as SABA monotherapy fails to treat the underlying chronic inflammation.

Consequently, the GINA guidelines propose a personalized management framework based on a continuous cycle of “assess, adjust, and review,” organized into two primary treatment tracks:

- **Track 1 (Preferred):** This track utilizes a combination low-dose inhaled corticosteroid (ICS)-formoterol inhaler as the sole reliever medication. In Steps 1–2, it is used as needed for symptom relief. In Steps 3–5, it serves as both daily maintenance and as-needed reliever therapy (MART). This approach ensures that patients receive an anti-inflammatory ICS dose with every reliever use, directly addressing inflammation when symptoms arise.
- **Track 2 (Alternative):** In this track, SABA is used as the reliever, but its use must be paired with an ICS-containing controller. For Step 1, this means taking an ICS dose whenever SABA is used. For Steps 2–5, it involves daily maintenance with ICS or an ICS-LABA combination.

GINA recommends Track 1 as its preferred treatment strategy for adults and adolescents with asthma. This approach is favored because it reduces the risk of severe exacerbations when compared with regimens using a SABA as a reliever. Furthermore, Track 1 may improve adherence, as patients use a single medication for both reliever and maintenance treatment across treatment steps. Finally, Track 1 is supported by evidence from four randomized controlled trials (SYGMA1 [12], SYGMA2 [13], PRACTICAL [14], and Novel START [15]), and by a systematic review and meta-analysis of all four studies for several outcomes [16]. However, both tracks feature five escalating steps of treatment intensity, with the objective of maintaining symptom control at the lowest effective dose. Treatment is tailored through regular reviews of symptom control, risk factors, and adherence, allowing for step-up or step-down adjustments. Step 5 includes specialist referral for the consideration of add-on therapies, such as biologic agents for severe asthma [1]. This individualized, risk-based strategy, particularly the move away from SABA-only treatment for even mild asthma, represents a significant advancement in asthma care.

The fundamental aims of asthma treatment are consistent globally, but their implementation in LMICs is often hindered by practical barriers. These challenges primarily involve the lack of affordable and accessible inhaled medicines and healthcare systems that prioritize acute treatment over long-term chronic care. Evidence shows that when provided to populations in LMICs, ICS-containing medications lead to remarkable decreases in mortality and morbidity [17]. However, access remains critically low. The availability of asthma treatments varies drastically among LMICs; some regions are limited to oral bronchodilators like salbutamol or theophylline, with only sporadic access to oral corticosteroids [18].

The scale of this issue was highlighted in a large 2011 survey of 52 countries [19]. It found that even a basic reliever like salbutamol was available in only half of public hospitals. Crucially, preventative ICS medications were available in less than 20% of public pharmacies and were completely absent in 14 of the surveyed nations. This means that the cornerstone of modern asthma therapy, which drastically reduces severe outcomes, is inaccessible to the vast majority of people with asthma globally. Forcing clinicians to manage asthma with only SABAs and oral corticosteroids is an unacceptable standard of care in the current era. As a pragmatic measure, Track 2 treatment can be considered in settings where the preferred Track 1 approach is not feasible due to these constraints on availability and affordability, even though it offers inferior protection against exacerbations.

## 2. Asthma Heterogeneity

### 2.1. Heritability, Key Loci, and Pharmacogenomics

Several studies have estimated the heritability of asthma to be as high as 55–90%, indicating a substantial genetic contribution to disease development [20]. The advent of genome-wide association studies (GWAS) has moved beyond familial observation to identify specific regions of the genome linked to asthma risk. These studies have pinpointed hundreds of associated loci, with the most robust and consistently replicated signal for childhood-onset asthma located on chromosome 17q12-q21 [21]. This region contains several key genes, including *ORMDL3* (involved in sphingolipid metabolism and inflammatory responses), *GSDMB* (gasdermin B), and *ZPBP2*, which are thought to play a role in epithelial function and immune regulation.

Despite this evidence, only a small fraction of the total estimated heritability is explained. The “missing heritability” [20] phenomenon suggests that asthma is not only a simple Mendelian disease but a polygenic one, arising from the combined small effects of many genes interacting with each other and with the environment. Furthermore, GWAS identifies statistical associations, not definitive causality, creating a “variant-to-function” gap that researchers are actively working to close [22]. To bridge this gap, researchers are employing advanced functional genomics techniques. For example, epigenetic mapping can identify whether disease-associated variants fall within regulatory regions of the genome in relevant cell types, while high-throughput CRISPR-based screening allows for the direct testing of how these variants impact gene function and expression. A major challenge is that most disease-associated variants are found in non-coding regions of the genome, suggesting their role is to regulate gene expression [22]. This regulatory function implies that a gene’s influence may only become apparent under specific circumstances, such as during a particular developmental stage or upon a specific environmental exposure.

Furthermore, it is estimated that genetic variation can account for up to 70% of the inter-individual variability in therapeutic response [23]:

- **Beta-agonists:** The response to SABA and LABA is significantly influenced by polymorphisms in the gene encoding their target, the β2-adrenergic receptor (*ADRB2*) [23,24]. The most studied polymorphism is Arg16Gly (rs1042713) where individuals homozygous for the Arginine-16 variant may exhibit a stronger and more rapid acute bronchodilator response to albuterol, but they may also experience a worsening of asthma control and an increased risk of exacerbations with regular, chronic use of SABAs [23]. Conversely, those with the Glycine-16 variant may have a different response profile.
- **Inhaled Corticosteroids (ICS):** Similarly, the efficacy of ICS is not uniform. Genetic variations in the corticotropin-releasing hormone receptor 1 gene (*CRHR1*) and the glucocorticoid receptor gene (*NR3C1*) are associated with differences in ICS response [23,24]. For example, certain haplotypes in the *CRHR1* gene are associated with a significantly greater improvement in lung function ($FEV_{1}$) in response to ICS treatment compared to other haplotypes [23,24].

Notably, the genetic architecture for childhood-onset and adult-onset asthma appears to be largely distinct. Fine-mapping analyses have revealed that the sets of likely causal genetic variants for these two major phenotypes have only about 16% overlap, strongly suggesting they are different diseases with different genetic origins [22]. It is important to clarify that while these pharmacogenomic associations are well-established in research settings, the testing for markers such as *ADRB2* or *CRHR1* variants is not currently recommended or routinely performed in clinical practice to guide asthma management. Their clinical utility remains investigational, pending further evidence to support a clear benefit for patient outcomes.

### 2.2. Early-Life Influences and Developmental Programming

The Developmental Origins of Health and Disease (DOHaD) hypothesis provides a framework for understanding how early-life environmental exposures program long-term susceptibility to chronic diseases like asthma [25]. This “critical window”, from gestation through infancy, is crucial for immune system development and maturation [26,27].

Several perinatal factors are established risks for developing asthma. Maternal asthma, maternal smoking during pregnancy, low birth weight, and prematurity are all consistently associated with impaired lung development and subsequent asthma risk [26,28]. Furthermore, interventions like early-life oxygen therapy increase risk, while breastfeeding has been shown to be a protective factor [28,29].

These risk factors often converge on key mechanisms involving immune education and the microbiome. The “hygiene hypothesis” posits that microbial exposure is necessary to calibrate the immune system away from its neonatal T-helper 2 (Th2) skew, a state that otherwise promotes allergic inflammation [27,30]. Evidence from farming environments, which are associated with lower asthma rates, supports this concept [31]. Conversely, early-life viral infections, particularly with RSV or HRV, can disrupt immune development and significantly increase the risk of persistent asthma [32,33].

The gut microbiome has been identified as a master regulator of this early immune programming through the “gut-lung axis” [27]. The establishment of a diverse gut microbiota is essential for systemic immune education, for example, relative abundance of the bacterial genera *Lachnospira*, *Veillonella*, *Faecalibacterium*, and *Rothia* was significantly decreased in children at risk of asthma [34]. Factors that disrupt this process, such as Cesarean delivery, antibiotic use, and formula feeding, are linked to gut dysbiosis and an elevated risk of asthma. In contrast, protective factors include vaginal birth, breastfeeding, and exposure to pets [27,34]. This connection is mechanistic, as gut bacteria produce metabolites like butyrate, propionate, and isovalerate and other short-chain fatty acids (SCFAs) [35] that systemically modulate inflammatory responses, including those in the lungs [34].

### 2.3. The Influence of Sex and Hormones

Asthma prevalence and severity exhibit significant sex-specific differences across the lifespan. Before puberty, asthma is more common and often more severe in boys. This pattern reverses during adolescence, and in adulthood, women demonstrate a higher prevalence, severity, and persistence of the disease [36,37,38]. This “pubertal switch” strongly implicates sex hormones as key modulators of asthma pathophysiology.

Female sex hormones, primarily estrogen and progesterone, are generally considered pro-inflammatory in the airways, promoting type 2 inflammation, eosinophil infiltration, and the upregulation of Th2 cytokines like IL-4 and IL-13 [37,38]. These hormonal effects manifest throughout a woman’s reproductive life:

- Menstrual Cycle: Approximately 20% of women experience “premenstrual asthma” (PMA), a cyclical worsening of symptoms during the perimenstrual phase [1]. PMA is more common in women with a higher BMI, a longer history of asthma, and aspirin-exacerbated respiratory disease [39].
- Puberty and Menopause: Early puberty is associated with a higher risk of developing asthma. Conversely, the onset of menopause is linked to an increased risk of current asthma and wheeze, with exacerbation rates appearing highest around this transition [40].
- Pregnancy: The course of asthma during pregnancy is variable, with approximately one-third of women experiencing worsening symptoms. These exacerbations are often linked to viral infections and poor adherence to medication. However, symptoms typically return to pre-pregnancy levels within three months postpartum, and asthma severity is not significantly affected when controller medications are used consistently [41,42,43].
- Hormonal Therapies: Three large observational studies [44,45,46] showed that hormonal contraceptives may have a protective effect against asthma incidence and severity, these findings were consistent across populations. Similarly, long-term hormone replacement therapy (HRT) in postmenopausal women has been associated with a lower incidence of asthma [47].

In contrast, testosterone, the primary male sex hormone, appears to have a protective effect. It can suppress Th2-driven inflammation and may inhibit airway remodeling, potentially explaining the frequent remission of childhood asthma in boys during puberty [37,38].

Sex also modifies the impact of other risk factors. Obesity-associated asthma, a severe phenotype, is significantly more prevalent in women. Furthermore, women may have greater susceptibility to the harmful effects of tobacco smoke, possibly due to estrogen-induced changes in toxin metabolism [39,48].

In summary, the clinical trajectory of asthma is fundamentally different between sexes. Childhood-onset, atopic asthma with a high chance of remission is more characteristic in males. In contrast, females are more likely to experience adult-onset, often non-atopic, and persistent disease influenced by hormonal cycles and a higher prevalence of comorbidities like obesity [49].

### 2.4. The Impact of Environmental Exposures

The environment continuously interacts with the immune system and plays an active role in asthma development, exacerbation, and chronic severity among susceptible individuals. The rising prevalence of asthma, particularly in developed nations, is largely attributed to changes in environmental exposures associated with urbanization and a “Westernized” lifestyle [50].

A diverse spectrum of environmental exposures can provoke airway inflammation and hyperresponsiveness. Key categories include:

- **Biologic Allergens:** These are classic triggers for atopic asthma and include indoor allergens (e.g., house dust mites, animal dander, molds) and outdoor allergens (e.g., pollens). The timing, dose, and type of allergen exposure influence sensitization and clinical phenotype.
- **Air Pollution:** Ambient air pollution is a major risk factor for both asthma development and exacerbation. Traffic-related air pollution (TRAP)—a mixture including particulate matter (PM_2.5_), nitrogen oxides (NO_*x*_), and ozone—is strongly associated with reduced lung growth and function, and the onset of childhood asthma [50]. These pollutants induce oxidative stress and disrupt airway epithelial integrity.
- **Occupational Exposures:** An estimated 5–20% of adult-onset asthma is attributable to occupational agents like industrial chemicals (isocyanates), dusts, and fumes [51,52]. Exposure risks often follow gendered employment patterns, though these differences may lessen as occupational roles evolve [49].

The impact of these exposures is profoundly modified by an individual’s genetic makeup, a concept known as gene–environment (GxE) interaction [50,53]. GxE interactions may help explain asthma’s heterogeneity and the “missing heritability” phenomenon, as a genetic predisposition may only manifest upon contact with a specific environmental trigger.

A well-documented example involves polymorphisms in genes encoding detoxification enzymes, such as glutathione S-transferase P1 (GSTP1). Variants that impair the enzyme’s ability to neutralize oxidative stress are linked to an increased asthma risk, but this association is strongest in children exposed to high levels of air pollution [54]. This illustrates that an individual’s response to their environment is shaped by their unique genetic profile, underscoring the importance of considering the lifelong “exposome” in clinical assessment.

### 2.5. Lifestyle Factors and Comorbidities

#### 2.5.1. Lifestyle’s Imprint

Lifestyle factors, including tobacco smoke exposure, diet, obesity, and physical activity, significantly modulate asthma control and severity.

Exposure to tobacco smoke is highly detrimental for individuals with asthma. Active smoking is associated with poorer symptom control, accelerated decline in lung function, increased exacerbation frequency, and higher asthma-related mortality [55,56,57]. Smoking induces a state of relative corticosteroid insensitivity, partly by promoting a neutrophilic, T2-low airway inflammation pattern that is less responsive to inhaled corticosteroids (ICS) [55]. Furthermore, exposure to secondhand and thirdhand smoke is a significant risk factor for asthma development in children and a trigger for exacerbations [58].

The parallel rise in obesity and asthma prevalence highlights a well-established link [59,60,61]. Obesity contributes to asthma severity through both mechanical and inflammatory mechanisms. Mechanically, central adiposity can restrict ventilation and reduce lung volumes. Metabolically, adipose tissue produces pro-inflammatory adipocytokines that contribute to low-grade systemic inflammation, often resulting in a severe, difficult-to-treat asthma phenotype [59]. In contrast, dietary patterns may offer protection. Diets rich in antioxidants, such as those that are plant-based, have been associated with reduced inflammation and improved lung health [62,63,64].

The role of exercise in asthma is complex. Physical exertion is a common trigger for exercise-induced bronchoconstriction (EIB), an acute airway narrowing diagnosed by a post-exercise drop in FEV1 of >10% [65,66]. Despite this, avoidance of physical activity is counterproductive. Regular exercise improves cardiovascular fitness and has been shown to enhance overall asthma control and reduce the frequency of EIB episodes. With appropriate management, most individuals with asthma can engage safely in physical activity [66,67].

#### 2.5.2. Comorbidities

Comorbid conditions are deeply integrated into the pathophysiology and clinical course of asthma, often driving poor control and exacerbating symptoms. A comprehensive assessment of these conditions is essential for effective asthma management.

- **Allergic Rhinitis (AR):** The strong association between AR and asthma has led to the “one airway, one disease” concept, which posits that they are different clinical manifestations of a single underlying systemic allergic process [68,69,70]. Both conditions share common triggers and inflammatory pathways. Affecting 60–80% of individuals with asthma, uncontrolled AR is a major risk factor for poor asthma control and increased exacerbation frequency [70,71]. A meta-analysis showed a decrease in asthma symptoms and AR after intranasal corticosteroid treatment [72]. Consequently, treatment of the upper airway is a critical component of managing the lower airway.
- **Gastroesophageal Reflux Disease (GERD):** GERD is a highly prevalent comorbidity in asthma, with its prevalence increasing with disease severity [73,74,75]. The relationship is bidirectional: acid reflux can trigger asthma symptoms via microaspiration or vagal nerve-mediated reflex bronchoconstriction. Conversely, the mechanics of labored breathing and certain asthma medications can worsen GERD. Unrecognized GERD is a common cause of treatment failure in patients with uncontrolled asthma [73]. Despite these relationships, randomized clinical trials have provided contrasting results. While some literature suggests that in asthmatics with symptoms of GERD, treatment may offer a small benefit to asthma-related quality of life and possibly exacerbations [76], there is no clear evidence supporting objective benefits in pulmonary function [77,78,79]
- **Psychological Conditions:** A significant bidirectional link exists between asthma and mental health disorders, particularly anxiety and depression [80,81]. There is an overlap in physical symptoms (e.g., shortness of breath, chest tightness), which can create diagnostic confusion. Furthermore, poor mental health is a primary driver of adverse asthma outcomes. Depression can lead to medication non-adherence, while anxiety may result in inappropriate healthcare utilization and medication overuse. There is emerging evidence suggesting that both cognitive behavioral therapy (CBT) and certain antidepressants may offer benefits in improving asthma control, primarily in patients who also experience psychological distress like anxiety and depression [82,83]. However, the current body of evidence is not yet robust enough to recommend these as standard treatments for all individuals with asthma.

The presence of these comorbidities highlights the necessity of a multidisciplinary approach to asthma care. Managing the lungs in isolation, without addressing concurrent rhinitis, gastroesophageal reflux, or psychological distress, is often an ineffective strategy for a significant subset of the asthma population.

## 3. Phenotypes and Endotypes

The clinical and biological heterogeneity of asthma necessitates a framework to guide diagnosis and treatment. This has led to the classification of asthma into “phenotypes” and “endotypes” [2,84,85]. A phenotype describes the observable characteristics of an individual’s asthma, while an endotype refers to the underlying molecular mechanism [2]. Linking phenotypes to endotypes is a central goal of precision medicine, enabling targeted therapy (Table 2).

**Figure 1:**
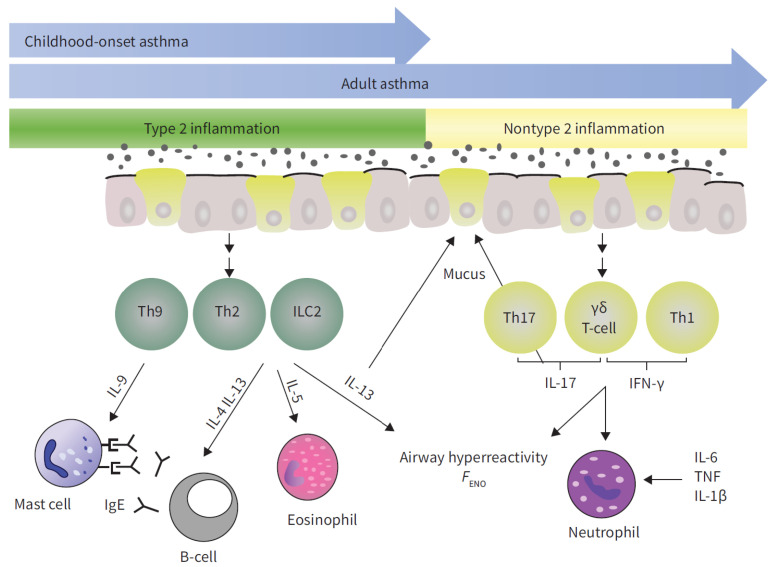
A schematic representation of inflammatory pathways in asthma: T2 inflammation is reported in green, non-T2 inflammation in yellow. F_ENO_: exhaled nitric oxide fraction; IFN: interferon; IL: interleukin; ILC2: type 2 innate lymphoid cell; Th: T-helper cell; TNF: tumor necrosis factor. Image taken from Chowdhury et al., 2021 [88].

Cluster analyses of large patient cohorts have identified several distinct asthma subtypes, with a general consensus defining two major endotypes, particularly in severe asthma (Figure 1) [86,87].

### 3.1. T2-High Endotype

T2-high endotype is driven by type 2 (T2) inflammation, mediated by cytokines such as IL-4, IL-5, and IL-13, which are produced by Th2 cells and ILC2s. This pathway leads to IgE production, eosinophilia, elevated fractional exhaled nitric oxide (FeNO), mucus hypersecretion, and airway hyperresponsiveness (AHR) [2]. T2-high asthma is typically responsive to corticosteroids and is the target of most available biologic therapies. Key associated phenotypes include:

- *Early-Onset Allergic Asthma*: This is the “classic” or “extrinsic” form of asthma, typically beginning in childhood. It is strongly associated with atopy and often coexists with other allergic conditions like eczema and allergic rhinitis.
- *Late-Onset Eosinophilic Asthma*: This phenotype typically manifests in adulthood, often in individuals without a significant history of childhood allergies. It is characterized by severe eosinophilia, which often require higher doses of ICS, and are prime candidates for biologic therapies that target T2 pathways.
- *Aspirin-Exacerbated Respiratory Disease (AERD)*: This is characterized by asthma, chronic rhinosinusitis with nasal polyposis (CRSwNP), and COX-1 inhibitor-induced respiratory reactions. Although the mechanisms underlying AERD are not fully elucidated, the ultimate result is severe persistent upper as well as lower airway disease with refractory CRSwNP and asthma.

### 3.2. T2-Low Endotype

T2-low endotype is characterized by non-T2 inflammation. This endotype often involves neutrophilic or paucigranulocytic patterns mediated by Th1 and Th17 cytokines (e.g., IFN-γ, TNF-α, IL-17A) [2,89]. Associated phenotypes include:

- *Obesity-Associated Asthma*: This phenotype is increasingly recognized, particularly in adult women. It presents a complex picture with multiple potential endotypes. Some patients exhibit T2-high inflammation, but many have a T2-low profile with minimal eosinophilia. The pathophysiology is multifactorial, involving mechanical effects of obesity on lung function, as well as systemic inflammation driven by pro-inflammatory mediators released from adipose tissue. This phenotype is notoriously difficult to treat and often shows a poor response to corticosteroids.
- *Very Late-Onset Asthma*: This is typically defined by onset after age 50 or 60, this phenotype is often associated with increased sputum neutrophilia linked to immunosenescence.
- *Smoking-associated Asthma*: This is a neutrophilic, steroid-resistant phenotype. It is distinct from asthma-COPD overlap (ACO), though ACO patients may have overlapping features and are sometimes eligible for biologic therapies [90].

T2-low endotype represents a major clinical challenge due to its characteristic resistance to corticosteroids and the lack of effective targeted therapies. Among emerging biological therapies described in the literature, key targets include components of the IL-23/IL-17 pathway, such as the monoclonal antibodies risankizumab (anti-IL-23) [91] and brodalumab (anti-IL-17R) [92]. To date, neither of these drugs has received approval for the treatment of severe asthma.

While this classification provides a rational basis for understanding asthma’s heterogeneity, significant overlap exists between groups, and individual patient profiles can evolve over time.

### 3.3. Biomarkers

The practical application of this framework hinges on the use of biomarkers to connect a patient’s clinical phenotype to their underlying endotype, which in turn guides therapeutic decisions and predicts treatment response, especially for severe asthma. This process is central to the practice of precision medicine in asthma care. For instance:

- **Serum Immunoglobulin E (IgE):** Elevated levels (>70–700 IU/mL) identify patients with an allergic asthma phenotype, making them suitable candidates for anti-IgE therapy like omalizumab [93,94].
- **Peripheral blood eosinophil count:** A readily available measure that serves as a surrogate for airway eosinophilia. Elevated levels (>150 × 10^3^ cell/μL serves as a key indicator for a T2-high, eosinophilic endotype. This biomarker predicts a favorable response to biologic therapies that target the IL-5 pathway, such as mepolizumab and benralizumab [93,94].
- **Fractional Exhaled Nitric Oxide (FeNO):** A non-invasive measure of IL-13-driven inflammation. High FeNO levels (>25 ppb) can predict a good response to ICS and certain biologics like dupilumab, which targets the shared IL-4 and IL-13 receptor [93,94].

By using these biomarkers, clinicians can deconstruct the heterogeneity of asthma in practice. They can move beyond symptom management to select a targeted biologic therapy that addresses the specific molecular pathway driving the patient’s disease, thereby personalizing treatment for severe asthma and improving outcomes.

## 4. The Future of Asthma Care

### 4.1. Targeted Therapies

For patients with severe asthma that remains uncontrolled despite high-dose ICS and LABA therapy, biologic agents represent the current standard of personalized medicine. These therapies target specific components of the type 2 (T2) inflammatory pathway.

The current arsenal of approved biologics includes several distinct classes (Table 3):

- **Anti-Immunoglobulin E (IgE) Therapy:** Omalizumab is a monoclonal antibody that binds to circulating IgE, preventing the activation of mast cells and basophils. It is indicated for severe allergic asthma in patients aged 6 years and older with confirmed allergen sensitization and elevated serum IgE [4,94].
- **Anti-Interleukin-5 (IL-5) Pathway Therapy:** This class targets the primary cytokine for eosinophil maturation and activation. Mepolizumab directly neutralizes IL-5. Benralizumab binds to the IL-5 receptor alpha subunit on eosinophils, blocking signaling and inducing eosinophil apoptosis via antibody-dependent cell-mediated cytotoxicity. These agents are indicated for severe eosinophilic asthma, identified by elevated blood eosinophil counts [4,94]. Approval ages vary by agent and region.
- **Anti-IL-4 and IL-13 Therapy:** Dupilumab targets the IL-4Rα subunit, which is common to the receptors for both IL-4 and IL-13, thereby inhibiting two key T2 cytokines. This dual blockade broadly suppresses T2 inflammation. It is indicated for patients with severe eosinophilic asthma or those with elevated fractional exhaled nitric oxide (FeNO) [4,94]. A notable side effect can be secondary hypereosinophilia [94].
- **Anti-Thymic Stromal Lymphopoietin (TSLP) Therapy:** Tezepelumab is an antibody targeting TSLP, an upstream “alarmin” cytokine released by the airway epithelium. By blocking TSLP, tezepelumab inhibits a broad range of downstream inflammatory cascades, including both T2 and some non-T2 pathways. It is therefore effective in a wider population of severe asthma patients, including those with lower eosinophil counts [4,94].

These therapies have demonstrated significant efficacy in reducing exacerbation rates, decreasing oral corticosteroid (OCS) dependence, and improving lung function and quality of life in appropriately selected patients. Many are also approved for comorbid T2-driven conditions, such as chronic rhinosinusitis with nasal polyposis (CRSwNP), supporting an integrated “one airway, one disease” treatment approach.

**Table 3:** Biologic Therapies for Severe Asthma: Targets, Indications, and Key Efficacy Outcomes. This table provides a concise summary of the current precision therapies available, linking them directly to the endotypes and biomarkers that guide their use [94]. OCS, Oral Corticosteroids; EU, European Union; USA, United States of America.

Biologic Agent	Target	Key Indication (Phenotype/Biomarkers)	Approved Age	Primary Efficacy Outcomes
**Omalizumab**	IgE	Severe allergic asthma (High IgE, perennial allergen sensitization)	≥6 years	Exacerbation reduction, OCS sparing
**Mepolizumab**	IL-5	Severe eosinophilic asthma (blood eosinophils ≥ 150/μL)	≥6 years	Exacerbation reduction, OCS sparing
**Benralizumab**	IL-5 receptor α	Severe eosinophilic asthma (blood eosinophils ≥ 300/μL)	≥18 years (EU) or ≥12 years (USA)	Exacerbation reduction, OCS sparing, rapid eosinophil depletion
**Dupilumab**	IL-4 receptor α	Severe eosinophilic/t2 asthma (eosinophils ≥ 150/μL or FeNO ≥ 25 ppb)	≥12 years	Exacerbation reduction, OCS sparing, improved lung function
**Tezepelumab**	TSLP	Severe asthma (broad indication, including T2-low)	≥12 years	Exacerbation reduction across eosinophil levels, improved lung function

### 4.2. The Multi-Omics Revolution

While biologic therapies represent the current application of precision medicine in asthma, future advancements are expected to arise from the integration of multi-omics approaches [95,96,97]. This systems-biology strategy involves the integrated analysis of large-scale datasets from multiple biological levels, rather than focusing on single data points in isolation. The objective is to construct a comprehensive molecular profile of each individual. Key layers of analysis include:

- **Genomics:** Identification of the complete set of genetic risk variants.
- **Epigenomics:** Mapping of chemical modifications to DNA (e.g., methylation) that alter gene expression in response to environmental factors.
- **Transcriptomics:** Profiling of gene expression (RNA) in relevant cells and tissues.
- **Proteomics:** Characterization of the full complement of cellular proteins.
- **Metabolomics:** Measurement of small-molecule metabolites reflecting real-time physiological status.
- **Microbiomics:** Analysis of the composition and function of the gut and airway microbial communities.

By integrating these large-scale “omics” datasets, researchers aim to move beyond the current reliance on single data points and construct a comprehensive molecular profile for each patient. The ultimate goal is to use these profiles to identify robust and predictive biomarker signatures that can more accurately guide treatment. In clinical practice, this could revolutionize patient care. For example, a multi-omics signature might identify a specific subgroup of patients with T2-low asthma who, despite having low eosinophil counts, would respond to a particular biologic. This would allow for a level of mechanistic precision not currently possible [96], enabling truly individualized treatment strategies and helping to solve the critical unmet need for effective therapies in non-type 2 asthma. This approach holds the potential to predict which specific biologic a patient will respond to, thereby maximizing efficacy and improving outcomes.

### 4.3. Unmet Needs

Despite the remarkable progress, significant gaps and challenges remain in asthma research and management. These unmet needs define the priorities for the future and reinforce the necessity of a personalized approach:

- **Therapies for non-T2 Asthma:** The development of effective, targeted therapies for T2-low asthma (e.g., neutrophilic, paucigranulocytic) remains a major challenge and a critical unmet need, as these patients are often resistant to both corticosteroids and the current biologics [3,98].
- **Predictive and Accessible Biomarkers:** While current biomarkers for T2 inflammation are useful, there is an urgent need for better, cheaper, and more accessible biomarkers that can accurately predict which specific biologic a patient will respond to, monitor treatment response, and identify patients with non-T2 disease [3,98].
- **Disease Modification and Remission:** Current asthma therapies are highly effective at controlling inflammation and symptoms, but they do not cure the disease. A paramount goal for future research is the development of disease-modifying therapies that can alter the natural history of asthma, prevent or reverse airway remodeling, and induce a state of sustained, treatment-free remission [3,98].
- **Primary Prevention:** The ultimate goal is to prevent asthma from developing in the first place. A deeper understanding of the gene–environment interactions and developmental programming that occur in the early-life “critical window” is essential for designing effective primary prevention strategies [3,98].
- **Bridging the Implementation Gap:** Even with existing knowledge, significant gaps remain in clinical practice, including high rates of misdiagnosis, poor inhaler technique, suboptimal medication adherence, and a failure to address comorbidities systematically [3,98]. Overcoming these barriers requires innovative and practical approaches. For instance, the implementation of structured care pathways could ensure that every patient receives a systematic assessment, guideline-based treatment, and integrated management of comorbidities. Furthermore, the burgeoning field of digital health offers powerful tools to tackle poor adherence. Digital adherence platforms, which include smart inhalers, companion mobile applications, and automated reminders, have been shown to improve medication adherence and asthma control by providing real-time feedback and support to patients. While challenges such as the digital divide and upfront costs must be considered, particularly in low- and middle-income settings, these strategies represent tangible solutions to close the gap between evidence and real-world practice.

## 5. Conclusions

The conceptualization of asthma has undergone a significant evolution, moving from the view of a single disease entity to its current understanding as a complex and heterogeneous syndrome. This shift represents a critical advancement in respiratory medicine.

The clinical expression of asthma in an individual is determined by a complex interplay of intrinsic and extrinsic factors. Genetic predisposition provides the foundational susceptibility, but its penetrance and expression are modulated throughout an individual’s life. Critical windows in early development, including perinatal events and the establishment of the gut microbiome, are pivotal in programming the long-term immune response. Furthermore, sex and hormonal fluctuations introduce significant biological variance, leading to distinct disease pathways and outcomes between males and females. The continuous interaction between an individual’s genetic background and environmental exposures, such as allergens and air pollution, is crucial in both initiating and perpetuating the disease. Lifestyle factors, including diet and smoking, along with the presence of comorbidities like chronic rhinosinusitis, gastroesophageal reflux, and psychological stress, further modify the disease course.

To manage this heterogeneity, the clinical framework now links observable phenotypes to their underlying molecular endotypes. The use of biomarkers is the critical bridge in this framework, providing the foundation for clinical decision-making. This process enables clinicians to move beyond symptom management and actively engage in treatment targeting and response prediction. For instance, a biomarker such as elevated blood eosinophils helps identify a patient with a T2-high endotype, which in turn predicts a favorable response to anti-IL-5 or anti-IL-5 receptor therapies. This ability to select a specific biologic therapy based on a patient’s molecular profile is the most significant clinical success of the precision approach, offering profound benefits for those with severe asthma.

The ongoing integration of multi-omics data promises to further refine this understanding, creating a more granular and systems-level portrait of each patient’s disease.

Despite these advances, significant challenges remain. The pathophysiology of certain subgroups, particularly non-T2 asthma, remains poorly defined, and there is an urgent need for reliable predictive biomarkers to guide therapy and monitor outcomes. The ultimate goals of achieving disease modification and primary prevention are not yet realized. Addressing these unmet needs requires a firm commitment to the principles of personalized medicine. Therefore, it is imperative to move beyond a uniform treatment paradigm. It is our opinion that future studies must adopt a systems-biology strategy, with a multi-omics approach that constructs a comprehensive molecular profile of each patient. This integrative methodology will require the use of advanced computational methods, including machine learning and artificial intelligence, to decipher the complex pathways linking genetic and environmental inputs to clinical disease. The explicit goal of this approach is to uncover novel disease mechanisms, identify more robust and predictive biomarker signatures, discover new therapeutic targets, and ultimately define patient subgroups with a level of mechanistic precision that is not currently possible. 

## Figures and Tables

**Table 2:** Key Asthma Phenotypes and Endotypes: Characteristics and Biomarkers. This table synthesizes the diverse factors contributing to asthma heterogeneity into clinically relevant groupings, linking observable features to underlying biology and the biomarkers used for their identification.

Phenotype (Endotype)	Key Clinical Features	Primary Inflammatory Pathway	Key Biomarkers
**Early-Onset Allergic** (T2-high)	Onset in childhood; strong atopic background (eczema, AR); often mild-to-moderate; generally ICS-responsive.	T2-high: IL-4, IL-5, IL-13 mediated.	High serum IgE, positive allergen skin tests, blood eosinophils, FeNO.
**Late-Onset Eosinophilic** (T2-high)	Onset in adulthood; less atopy; often severe; associated with CRSwNP, AERD.	T2-high: predominantly IL-5, IL-13 mediated.	high blood/sputum eosinophils, high FeNO.
**Aspirin-Exacerbated Respiratory Disease (AERD)** (T2-high)	Onset in adulthood; chronic rhinosinusitis with nasal polyposis (CRSwNP) and COX-1 inhibitor-induced respiratory reactions	T2-high: predominantly IL-5, IL-13 mediated	High blood/sputum eosinophils, high FeNO.
**Obesity-Associated** (T2-low)	Adult-onset, more common in females; variable severity; often poor ICS response.	T2-low; systemic inflammation from adipose tissue.	Variable; may have elevated CRP, leptin.
**Very Late-Onset Asthma** (T2-low)	Onset in adulthood (>50 or >60 years): corticosteroid-resistant.	T2-low: IL-17, TNF-α mediated.	Sputum neutrophils. Lack of T2 biomarkers.
**Smoking-associated Asthma** (T2-low)	Associated with smoking. Corticosteroid-resistant.	T2-low: IL-17, TNF-α mediated.	Sputum neutrophils. Lack of T2 biomarkers

## Data Availability

Due to privacy and ethical considerations, the authors can provide the dataset supporting the reported results upon request. Interested parties can contact the correspondence author directly via email to request access.

## References

[1] (2025). Global Strategy for Asthma Management and Prevention. https://ginasthma.org/2025-gina-strategy-report/.

[2] Kuruvilla M.E., Lee F.E.H., Lee G.B. (2019). Understanding Asthma Phenotypes, Endotypes, and Mechanisms of Disease. Clin. Rev. Allergy Immunol..

[3] Busse W.W., Kraft M. (2022). Current Unmet Needs and Potential Solutions to Uncontrolled Asthma. Eur. Respir. Rev..

[4] Global Strategy for Asthma Management and Prevention (2024). Difficult-to-Treat & Severe Asthma in Adolescent and Adult Patients. https://ginasthma.org/wp-content/uploads/2024/11/GINA-Severe-Asthma-Guide-2024-WEB-WMS.pdf.

[5] World Health Organization Non Communicable Diseases. https://www.who.int/news-room/fact-sheets/detail/noncommunicable-diseases.

[6] World Health Organization Global Health Estimates 2021: Deaths by Cause, Age, Sex, by Country and by Region, 2000–2021. https://www.who.int/data/gho/data/themes/mortality-and-global-health-estimates/ghe-leading-causes-of-death.

[7] GBD 2019 Asthma Collaborators (2022). Global, regional, and national burden of asthma from 1990 to 2019: A systematic analysis for the Global Burden of Disease Study 2019. Lancet Respir. Med..

[8] Yuan L., Tao J., Wang J., She W., Zou Y., Li R., Ma Y., Sun C., Bi S., Wei S. (2025). Global, Regional, National Burden of Asthma from 1990 to 2021, with Projections of Incidence to 2050: A Systematic Analysis of the Global Burden of Disease Study 2021. eClinicalMedicine.

[9] Centers for Disease Control and Prevention (2023). FastStats—Asthma. https://www.cdc.gov/nchs/fastats/asthma.htm.

[10] Palange P., Rohde G. (2019). ERS Handbook of Respiratory Medicine.

[11] Martinez F.D. (2010). The Paradoxes of Asthma. Lancet.

[12] O’Byrne P.M., FitzGerald J.M., Bateman E.D., Barnes P.J., Zhong N., Keen C., Jorup C., Lamarca R., Ivanov S., Reddel H.K. (2018). Inhaled Combined Budesonide–Formoterol as Needed in Mild Asthma. N. Engl. J. Med..

[13] Bateman E.D., Reddel H.K., O’Byrne P.M., Barnes P.J., Zhong N., Keen C., Jorup C., Lamarca R., Siwek-Posluszna A., FitzGerald J.M. (2018). As-Needed Budesonide–Formoterol versus Maintenance Budesonide in Mild Asthma. N. Engl. J. Med..

[14] Hardy J., Baggott C., Fingleton J., Reddel H.K., Hancox R.J., Harwood M., Corin A., Sparks J., Hall D., Sabbagh D. (2019). Budesonide-Formoterol Reliever Therapy versus Maintenance Budesonide plus Terbutaline Reliever Therapy in Adults with Mild to Moderate Asthma (PRACTICAL): A 52-Week, Open-Label, Multicentre, Superiority, Randomised Controlled Trial. Lancet.

[15] Beasley R., Holliday M., Reddel H.K., Braithwaite I., Ebmeier S., Hancox R.J., Harrison T., Houghton C., Oldfield K., Papi A. (2019). Controlled Trial of Budesonide–Formoterol as Needed for Mild Asthma. N. Engl. J. Med..

[16] Crossingham I., Turner S., Ramakrishnan S., Fries A., Gowell M., Yasmin F., Richardson R., Webb P., O’Boyle E., Hinks T.S. (2021). Combination Fixed-Dose Beta Agonist and Steroid Inhaler as Required for Adults or Children with Mild Asthma. Cochrane Database Syst. Rev..

[17] Suissa S., Ernst P. (2001). Inhaled Corticosteroids: Impact on Asthma Morbidity and Mortality. J. Allergy Clin. Immunol..

[18] Mortimer K., Masekela R., Ozoh O.B., Bateman E.D., Nantanda R., Yorgancıoğlu A.A., Chakaya J., Reddel H.K. (2022). The Reality of Managing Asthma in Sub-Saharan Africa—Priorities and Strategies for Improving Care. J. Pan Afr. Thorac. Soc..

[19] Babar Z.U.D., Lessing C., Mace C., Bissell K. (2013). The Availability, Pricing and Affordability of Three Essential Asthma Medicines in 52 Low- and Middle-Income Countries. PharmacoEconomics.

[20] Hernandez-Pacheco N., Pino-Yanes M., Flores C. (2019). Genomic Predictors of Asthma Phenotypes and Treatment Response. Front. Pediatr..

[21] Wan Y.I., Shrine N.R.G., Soler Artigas M., Wain L.V., Blakey J.D., Moffatt M.F., Bush A., Chung K.F., Cookson W.O.C.M., Strachan D.P. (2012). Genome-Wide Association Study to Identify Genetic Determinants of Severe Asthma. Thorax.

[22] Zhong X., Mitchell R., Billstrand C., Thompson E.E., Sakabe N.J., Aneas I., Salamone I.M., Gu J., Sperling A.I., Schoettler N. (2025). Integration of Functional Genomics and Statistical Fine-Mapping Systematically Characterizes Adult-Onset and Childhood-Onset Asthma Genetic Associations. Genome Med..

[23] Lima J.J., Blake K.V., Tantisira K.G., Weiss S.T. (2009). Pharmacogenetics of Asthma. Curr. Opin. Pulm. Med..

[24] Blake K., Lima J. (2015). Pharmacogenomics of Long-Acting β_2_-Agonists. Expert Opin. Drug Metab. Toxicol..

[25] Langley-Evans S.C., McMullen S. (2010). Developmental Origins of Adult Disease. Med Princ. Pract..

[26] Martinez F.D. (2007). Genes, Environments, Development and Asthma: A Reappraisal. Eur. Respir. J..

[27] Boulund U., Thorsen J., Trivedi U., Tranæs K., Jiang J., Shah S.A., Stokholm J. (2025). The Role of the Early-Life Gut Microbiome in Childhood Asthma. Gut Microbes.

[28] Kim A., Lim G., Oh I., Kim Y., Lee T., Lee J. (2018). Perinatal Factors and the Development of Childhood Asthma. Ann. Allergy Asthma Immunol. Off. Publ. Am. Coll. Allergy Asthma Immunol..

[29] Xue M., Dehaas E., Chaudhary N., O’Byrne P., Satia I., Kurmi O.P. (2021). Breastfeeding and Risk of Childhood Asthma: A Systematic Review and Meta-Analysis. ERJ Open Res..

[30] Strachan D.P. (1989). Hay Fever, Hygiene, and Household Size. BMJ.

[31] von Mutius E., Vercelli D. (2010). Farm Living: Effects on Childhood Asthma and Allergy. Nat. Rev. Immunol..

[32] Murrison L.B., Brandt E.B., Myers J.B., Hershey G.K.K. (2019). Environmental Exposures and Mechanisms in Allergy and Asthma Development. J. Clin. Investig..

[33] Food and Drug Administration (2018). Asthma: The Hygiene Hypothesis. https://www.fda.gov/vaccines-blood-biologics/consumers-biologics/asthma-hygiene-hypothesis.

[34] Arrieta M.C., Stiemsma L.T., Dimitriu P.A., Thorson L., Russell S., Yurist-Doutsch S., Kuzeljevic B., Gold M.J., Britton H.M., Lefebvre D.L. (2015). Early Infancy Microbial and Metabolic Alterations Affect Risk of Childhood Asthma. Sci. Transl. Med..

[35] Arpaia N., Campbell C., Fan X., Dikiy S., van der Veeken J., deRoos P., Liu H., Cross J.R., Pfeffer K., Coffer P.J. (2013). Metabolites Produced by Commensal Bacteria Promote Peripheral Regulatory T-Cell Generation. Nature.

[36] Almqvist C., Worm M., Leynaert B., for the Working Group of GA2LEN WP 2.5 ‘Gender’ (2008). Impact of Gender on Asthma in Childhood and Adolescence: A GA2LEN Review. Allergy.

[37] Zein J.G., Denson J.L., Wechsler M.E. (2019). Asthma over the Adult Life Course. Clin. Chest Med..

[38] Zein J.G., Erzurum S.C. (2015). Asthma Is Different in Women. Curr. Allergy Asthma Rep..

[39] Brenner B.E., Holmes T.M., Mazal B., Camargo C.A. (2005). Relation between Phase of the Menstrual Cycle and Asthma Presentations in the Emergency Department. Thorax.

[40] McCleary N., Nwaru B.I., Nurmatov U.B., Critchley H., Sheikh A. (2018). Endogenous and exogenous sex steroid hormones in asthma and allergy in females: A systematic review and meta-analysis. J. Allergy Clin. Immunol..

[41] Schatz M., Harden K., Forsythe A., Chilingar L., Hoffman C., Sperling W., Zeiger R.S. (1988). The Course of Asthma during Pregnancy, Post Partum, and with Successive Pregnancies: A Prospective Analysis. J. Allergy Clin. Immunol..

[42] Yung J.A., Fuseini H., Newcomb D.C. (2018). Hormones, sex, and asthma. Ann. Allergy Asthma Immunol..

[43] Belanger K., Hellenbrand M.E., Holford T.R., Bracken M. (2010). Effect of Pregnancy on Maternal Asthma Symptoms and Medication Use. Obstet. Gynecol..

[44] Nwaru B.I., Sheikh A. (2015). Hormonal contraceptives and asthma in women of reproductive age: Analysis of data from serial national Scottish Health Surveys. J. R. Soc. Med..

[45] Nwaru B.I., Pillinger R., Tibble H., Shah S.A., Ryan D., Critchley H., Price D., Hawrylowicz C.M., Simpson C.R., Soyiri I.N. (2020). Hormonal contraceptives and onset of asthma in reproductive-age women: Population-based cohort study. J. Allergy Clin. Immunol..

[46] Nwaru B.I., Tibble H., Shah S.A., Pillinger R., McLean S., Ryan D.P., Critchley H., Price D.B., Hawrylowicz C.M., Simpson C.R. (2021). Hormonal contraception and the risk of severe asthma exacerbation: 17-year population-based cohort study. Thorax.

[47] Troisi R.J., Willett W.C., Weiss S.T., Trichopoulos D., Rosner B., Speizer F.E. (1995). A prospective study of diet and adult-onset asthma. Am. J. Respir. Crit. Care Med..

[48] Gibbs C.J., Coutts I.I., Lock R., Finnegan O.C., White R.J. (1984). Premenstrual Exacerbation of Asthma. Thorax.

[49] Marinelli A., Dragonieri S., Portacci A., Quaranta V.N., Carpagnano G.E. (2025). Reconsidering Gender in Asthma: Is It All About Sex? A Perspective Review. J. Clin. Med..

[50] Mallol J., Crane J., von Mutius E., Odhiambo J., Keil U., Stewart A., ISAAC Phase Three Study Group (2013). The International Study of Asthma and Allergies in Childhood (ISAAC) Phase Three: A Global Synthesis. Allergol. Immunopathol..

[51] Chan-Yeung M., Malo J.L. (1995). Occupational Asthma. N. Engl. J. Med..

[52] Mapp C.E., Boschetto P., Maestrelli P., Fabbri L.M. (2005). Occupational Asthma. Am. J. Respir. Crit. Care Med..

[53] von Mutius E. (2009). Gene-Environment Interactions in Asthma. J. Allergy Clin. Immunol..

[54] Fryer A.A., Bianco A., Hepple M., Jones P.W., Strange R.C., Spiteri M.A. (2000). Polymorphism at the Glutathione S-Transferase GSTP1 Locus. Am. J. Respir. Crit. Care Med..

[55] Chalmers G.W., Macleod K.J., Little S.A., Thomson L.J., McSharry C.P., Thomson N.C. (2002). Influence of Cigarette Smoking on Inhaled Corticosteroid Treatment in Mild Asthma. Thorax.

[56] Chalmers G.W., MacLeod K.J., Thomson L., Little S.A., McSharry C., Thomson N.C. (2001). Smoking and Airway Inflammation in Patients With Mild Asthma. Chest.

[57] Thomson N.C., Chaudhuri R., Livingston E. (2004). Asthma and Cigarette Smoking. Eur. Respir. J..

[58] He Z., Wu H., Zhang S., Lin Y., Li R., Xie L., Li Z., Sun W., Huang X., Zhang C.J.P. (2020). The Association between Secondhand Smoke and Childhood Asthma: A Systematic Review and Meta-Analysis. Pediatr. Pulmonol..

[59] Beuther D.A., Weiss S.T., Sutherland E.R. (2006). Obesity and Asthma. Am. J. Respir. Crit. Care Med..

[60] Miethe S., Karsonova A., Karaulov A., Renz H. (2020). Obesity and Asthma. J. Allergy Clin. Immunol..

[61] Peters U., Dixon A.E., Forno E. (2018). Obesity and Asthma. J. Allergy Clin. Immunol..

[62] Devereux G., Seaton A. (2005). Diet as a Risk Factor for Atopy and Asthma. J. Allergy Clin. Immunol..

[63] Julia V., Macia L., Dombrowicz D. (2015). The Impact of Diet on Asthma and Allergic Diseases. Nat. Rev. Immunol..

[64] McKeever T.M., Britton J. (2004). Diet and Asthma. Am. J. Respir. Crit. Care Med..

[65] Anderson S.D., Kippelen P. (2005). Exercise-Induced Bronchoconstriction: Pathogenesis. Curr. Allergy Asthma Rep..

[66] Parsons J.P., Hallstrand T.S., Mastronarde J.G., Kaminsky D.A., Rundell K.W., Hull J.H., Storms W.W., Weiler J.M., Cheek F.M., Wilson K.C. (2013). An Official American Thoracic Society Clinical Practice Guideline: Exercise-Induced Bronchoconstriction. Am. J. Respir. Crit. Care Med..

[67] Lucas S.R., Platts-Mills T.A.E. (2005). Physical Activity and Exercise in Asthma: Relevance to Etiology and Treatment. J. Allergy Clin. Immunol..

[68] Brożek J.L., Bousquet J., Agache I., Agarwal A., Bachert C., Bosnic-Anticevich S., Brignardello-Petersen R., Canonica G.W., Casale T., Chavannes N.H. (2017). Allergic Rhinitis and Its Impact on Asthma (ARIA) Guidelines—2016 Revision. J. Allergy Clin. Immunol..

[69] Grossman J. (1997). One Airway, One Disease. Chest.

[70] Giavina-Bianchi P., Takejima P., Kalil J., Agondi R.C. (2016). United Airway Disease: Current Perspectives. J. Asthma Allergy.

[71] Corren J. (1998). The Impact of Allergic Rhinitis on Bronchial Asthma. J. Allergy Clin. Immunol..

[72] Lohia S., Schlosser R.J., Soler Z.M. (2013). Impact of Intranasal Corticosteroids on Asthma Outcomes in Allergic Rhinitis: A Meta-Analysis. Allergy.

[73] Harding S.M. (1999). Gastroesophageal Reflux and Asthma: Insight into the Association. J. Allergy Clin. Immunol..

[74] McCallister J.W., Parsons J.P., Mastronarde J.G. (2011). The Relationship between Gastroesophageal Reflux and Asthma: An Update. Ther. Adv. Respir. Dis..

[75] Leggett J.J., Johnston B.T., Mills M., Gamble J., Heaney L.G. (2005). Prevalence of Gastroesophageal Reflux in Difficult Asthma. Chest.

[76] Littner M.R., Leung F.W., Ballard E.D., Huang B., Samra N.K., Lansoprazole Asthma Study Group (2005). Effects of 24 Weeks of Lansoprazole Therapy on Asthma Symptoms, Exacerbations, Quality of Life, and Pulmonary Function in Adult Asthmatic Patients with Acid Reflux Symptoms. Chest.

[77] Mastronarde J.G., Anthonisen N.R., Castro M., Holbrook J.T., Leone F.T., Teague W.G., Wise R.A. (2009). Efficacy of Esomeprazole for Treatment of Poorly Controlled Asthma. N. Engl. J. Med..

[78] Kiljander T.O., Harding S.M., Field S.K., Stein M.R., Nelson H.S., Ekelund J., Illueca M., Beckman O., Sostek M.B. (2006). Effects of Esomeprazole 40 Mg Twice Daily on Asthma: A Randomized Placebo-Controlled Trial. Am. J. Respir. Crit. Care Med..

[79] Kiljander T.O., Junghard O., Beckman O., Lind T. (2010). Effect of Esomeprazole 40 Mg Once or Twice Daily on Asthma: A Randomized, Placebo-Controlled Study. Am. J. Respir. Crit. Care Med..

[80] Alonso J., de Jonge P., Lim C.C.W., Aguilar-Gaxiola S., Bruffaerts R., Caldas-de-Almeida J.M., Liu Z., O’Neill S., Stein D.J., Viana M.C. (2014). Association between Mental Disorders and Subsequent Adult Onset Asthma. J. Psychiatr. Res..

[81] Richardson L.P., Lozano P., Russo J., McCauley E., Bush T., Katon W. (2006). Asthma Symptom Burden: Relationship to Asthma Severity and Anxiety and Depression Symptoms. Pediatrics.

[82] Kew K.M., Nashed M., Dulay V., Yorke J. (2016). Cognitive Behavioural Therapy (CBT) for Adults and Adolescents with Asthma. Cochrane Database Syst. Rev..

[83] Gajewski A.J., Palka J.M., Raitt J.M., Agarwal C.D., Khan D.A., Kao C.H., Brown E.S. (2023). Association of Serotonin Reuptake Inhibitors with Asthma Control. Allergy Asthma Proc..

[84] Anderson G.P. (2008). Endotyping Asthma: New Insights into Key Pathogenic Mechanisms in a Complex, Heterogeneous Disease. Lancet.

[85] Lambrecht B.N., Hammad H. (2015). The Immunology of Asthma. Nat. Immunol..

[86] Loza M.J., Djukanovic R., Chung K.F., Horowitz D., Ma K., Branigan P., Barnathan E.S., Susulic V.S., Silkoff P.E., Sterk P.J. (2016). Validated and Longitudinally Stable Asthma Phenotypes Based on Cluster Analysis of the ADEPT Study. Respir. Res..

[87] Moore W.C., Meyers D.A., Wenzel S.E., Teague W.G., Li H., Li X., D’Agostino R., Castro M., Curran-Everett D., Fitzpatrick A.M. (2010). Identification of Asthma Phenotypes Using Cluster Analysis in the Severe Asthma Research Program. Am. J. Respir. Crit. Care Med..

[88] Chowdhury N.U., Guntur V.P., Newcomb D.C., Wechsler M.E. (2021). Sex and gender in asthma. Eur. Respir. Rev..

[89] Nadif R., Siroux V., Boudier A., Le Moual N., Just J., Gormand F., Pison C., Matran R., Pin I. (2016). Blood granulocyte patterns as predictors of asthma phenotypes in adults from the EGEA study. Eur. Respir. J..

[90] Global Initiative for Asthma and Global Initiative for Chronic Obstructive Lung Disease (2015). Diagnosis of Diseases of Chronic Airflow Limitation: Asthma COPD and Asthma-COPD Overlap Syndrome (ACOS). https://goldcopd.org/wp-content/uploads/2016/04/GOLD_ACOS_2015.pdf.

[91] Brightling C.E., Nair P., Cousins D.J., Louis R., Singh D. (2021). Risankizumab in severe asthma—A phase 2a, placebo-controlled trial. N. Engl. J. Med..

[92] Busse W.W., Holgate S., Kerwin E., Chon Y., Feng J., Lin J., Lin S.L. (2013). Randomized, double-blind, placebo-controlled study of brodalumab, a human anti-IL-17 receptor monoclonal antibody, in moderate to severe asthma. Am. J. Respir. Crit. Care Med..

[93] Tiotiu A. (2018). Biomarkers in Asthma: State of the Art. Asthma Res. Pract..

[94] Brusselle G.G., Koppelman G.H. (2022). Biologic Therapies for Severe Asthma. New Engl. J. Med..

[95] Gautam Y., Johansson E., Mersha T.B. (2022). Multi-Omics Profiling Approach to Asthma: An Evolving Paradigm. J. Pers. Med..

[96] Tyler S.R., Bunyavanich S. (2019). Leveraging—Omics for Asthma Endotyping. J. Allergy Clin. Immunol..

[97] Zhang W., Zhang Y., Li L., Chen R., Shi F. (2024). Unraveling Heterogeneity and Treatment of Asthma through Integrating Multi-Omics Data. Front. Allergy.

[98] Caminati M., Vaia R., Furci F., Guarnieri G., Senna G. (2021). Uncontrolled Asthma: Unmet Needs in the Management of Patients. J. Asthma Allergy.

